# Enhancing translational research impact through collaborative process innovation

**DOI:** 10.1017/cts.2025.10186

**Published:** 2025-11-03

**Authors:** Marisha E. Palm, Sharon Phares, Gigi Hirsch

**Affiliations:** 1 Institute for Clinical Research and Health Policy Studies, Tufts Medical Center, Boston, MA, USA; 2 Tufts Clinical and Translational Science Institute, Tufts University, Boston, MA, USA

**Keywords:** Biomedical innovation, collaborative innovation, engagement, innovation stewardship, translational science

## Abstract

Translational science methods often fall short due to the complexity of the healthcare delivery environment. We developed a methodology that involves multiple interest holders working within a pre-competitive consortium to develop solutions to translational barriers. The methodology supports innovative collaboration in a stepwise fashion: elucidating challenges, designing solutions, enabling implementation, monitoring, learning, disseminating, and catalyzing. Cases that benefit most from a structured collaborative methodology are those where diverse needs require elucidation and alignment. Application of the methodology to develop regulatory, clinical, and business innovations has shown the importance of an innovation facilitator and the capacity-building potential of collective skill enhancement.

## Introduction

Biomedical science has advanced in profound ways over the last couple of decades, providing earlier diagnosis, better treatments, and allowing people to live healthier and longer lives. While biomedical innovation is crucial for progress, it also presents challenges that must be addressed to support the successful translation of research into health impact. Current methods often fall short due to the complex and dynamic nature of the real-world healthcare delivery environment [1,2]. This leads to failure at the final hurdle: translating health research to improved population health [3,4]. Reasons for this failure include real-world gaps in evidence, reimbursement challenges [5], policies that slow innovation, social influencers of health [6], and limited system capacity. Developing effective and sustainable solutions for these challenges often requires input from multiple interest holder groups and buy-in across organizations.

The NEW Drug Development ParadIGmS (NEWDIGS) consortium has developed a methodology that supports systems thinking and collaborative innovation among multiple interest holders [7–9]. The range of interest holders engaged include patients, clinicians, payers, life science companies, regulators, and investors, among others. Over the last 16 years, this methodology has been applied to many different complex healthcare challenges. Although the methodology is broadly applicable, NEWDIGS has worked mainly in the later stages of translation, focusing on features of the healthcare system that slow or prevent appropriate, timely, and equitable patient access to drug therapies. It has concentrated specifically on challenges that benefit from an external innovation environment that supports pre-competitive collaboration across organizational and interest holder silos.

In this manuscript, we illustrate the application of the NEWDIGS’ methodology using three case studies where the method has been used to develop scalable solutions to complex translational challenges in biomedical innovation.

## Newdigs Methodology

**Figure 1.**
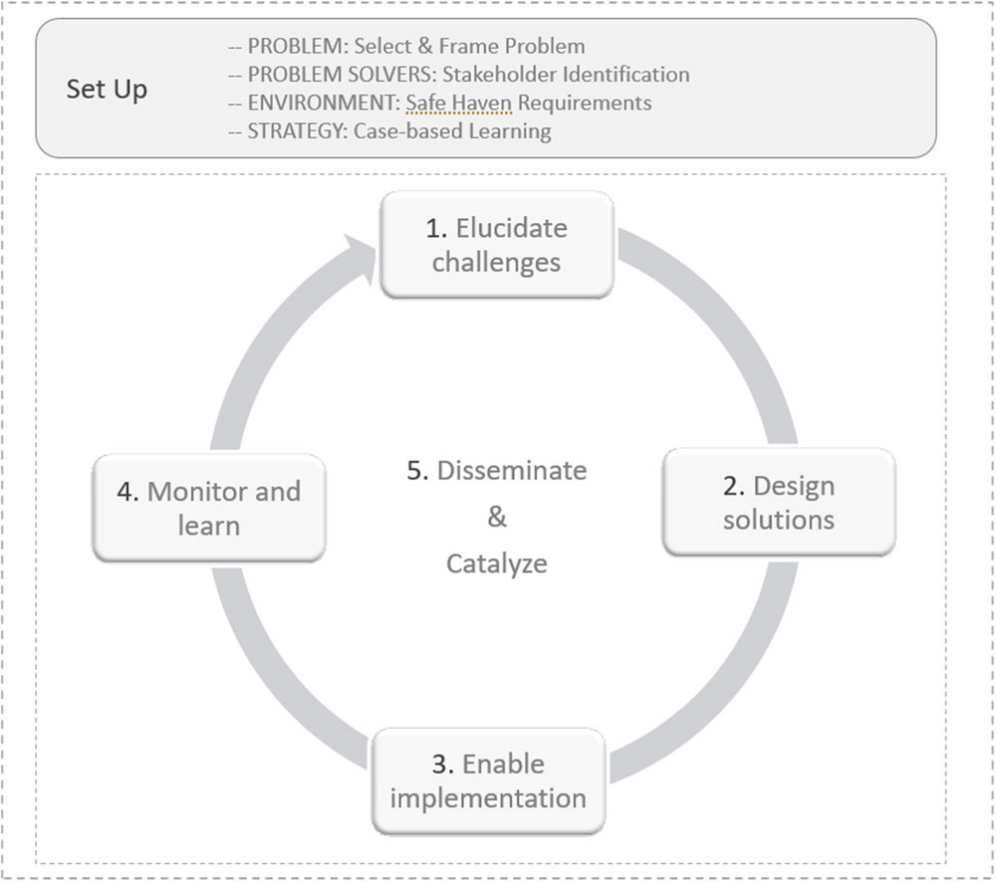
Overview including foundational setup activities and the five collaborative innovation process steps (Table 2).

NEWDIGS’ stepwise methodology for translating emerging science into real-world health impact is applied in an iterative cycle and illustrated in Figure 1.

**Table 1.** Activities to consider in building a safe haven innovation

Action	Purpose (assumption/reality)	Practical enablers
Clearly define the purpose of the collaboration	Interest holders often assumed the goal was consensus	Explicitly state that participants are not officially representing their employer organization in discussions Encourage participants to draw from all of their experiences, not just their current role Use of the Chatham House rule [12] to foster candid dialog
	The goal was candid dialog to explore a broad range of possibilities as components of a solution	
Foster creative solution design among interest holders	Interest holders may want to get to the “right” solution first	Share that it is okay for participants to feel lost at times in the creative process Instead of stating that a proposed solution will not work, offer ideas to improve it Structure design sessions in ways that support alignment across interest holders and organizations to develop solutions
	More often system solutions have multiple components that must be tailored to context	
Mitigate risks that may constrain collaboration value	Interest holders are sometimes more comfortable innovating within their own organizations	Clearly communicate Rules of Engagement on competition, conflicts of interest Institute non-disclosure agreements when appropriate Involve a strong facilitator to keep work toward collaborative solutions moving forward
	Some challenges require external collaboration with interest holders that may be competitors, and this process must be managed	

**Table 2.** Overview of NEWDIGS setup and collaborative innovation process for each project

Setup phase	Adaptive licensing	LEAPS – APoC	FoCUS – ORBM
**Problem**	The traditional one-size-fits-all model of regulatory decision-making is a binary go/no go decision at a single point in the innovation lifecycle. This can delay timely product access for patients who may need it most. The goal was to evaluate and refine a previously published model involving a more flexible, staged approach to regulatory approvals, and enable piloting in global settings.	Systematic learning about drug therapies ends at the point of regulatory approval, despite continued uncertainties about their safety, efficacy, and effectiveness in real-world use. The goal was to explore a novel approach to planning, production, and use of real-world evidence to accelerate regimen optimization at scale in patient care.	Financial risks associated with emerging durable cell and gene therapies threatened access for patients in need. The goal was to develop payment innovations that ensure access for patients and sustainability for the system.
**Problem solvers** *	Global regulators, health technology assessment bodies, and payers.	Pharmacoepidemiologists, clinical trialists, health economists, outcomes researchers, rheumatologists, and patients with rheumatoid arthritis.	Representatives of all major US public and private payer segments, pharmacists from integrated delivery systems, and investors.
**Environment**	A safe haven was created for all projects and the case studies within them. See Table 1 for further details.		
**Strategy**	A portfolio of 13 case studies, each focused on an individual product under development in the global industry to explore design and implementation of the new, generalizable regulatory model.	Case study of one selected disease (Rheumatoid Arthritis) with significant evidence gaps that impeded regimen optimization across the patient journey as context for design of new infrastructure for integrating research and care.	Case studies focused on three specific disease/product class pairs where access was threatened by financial risks, which required characterization, and the development of new payment models.
**Innovation process**	**Adaptive licensing**	**LEAPS – APoC**	**FoCUS – ORBM**
**Elucidate problem**	Identified potential benefits and risks of the proposed new model of “adaptive licensing” for each interest holder.	Identified major decision point in Rheumatoid Arthritis clinical guidelines where better real-world evidence could significantly improve clinical outcomes.	Distilled the key financial risks of durable cell and gene therapies into actuarial, performance uncertainty, and payment timing. Identified actuarial risk and execution challenges uniquely faced by small insurers.
**Design solutions**	Explored application of the Adaptive Licensing paradigm in a series of case studies (i.e., drug therapies) to develop a generalizable set of principles about how to apply it.	Designed an Adaptive Point-of-Care (APoC) platform that is embedded in clinical decision-making structured as a prospective study designed for continuous learning and improvement. Explored impact of solution on all interest holders.	Developed model of Orphan Reinsurance and Benefit Manager (ORBM), an innovative solution for US payers that integrates healthcare financing and management to increase consistency, pool risk, and create operational efficiencies.
**Enable implementation**	Identified and explored implementation barriers and potential solutions such as process and policy innovations. Evaluating feasibility and critical success drivers for pilots across different global jurisdictions.	Considered implementation barriers and enablers, including ways to align incentives across interest holders and resourcing requirements for this new infrastructure to ensure scalability and sustainability.	Explored potential adoption barriers, and identified intermediaries that could provide carve-outs in return for a capitated payment based on the overall size of the primary payer’s plan.

NEWDIGS =NEW Drug Development ParadIGmS; LEAPS = Learning Ecosystems Accelerator for Patient-Centered Sustainable Innovation; APoC = Adaptive Point of Care; FoCUS = Financing and Reimbursement of Cures in the US; ORBM = Orphan Reinsurer and Benefit Manager.

* Every project included patients or patient advocates, relevant providers, payers, and industry partners.

For each challenge undertaken, setup activities including selecting and framing the problem, identifying and engaging interest holders, building a safe haven, and developing a case-based strategy for learning, were key success drivers.

### Selecting and framing the problem

To select translational problems, we focused on challenges that shared specific characteristics: our “rules of three.” First, solutions required three or more interest holders; if the challenge could be successfully addressed by one or two organizations, we determined that a consortium approach was not needed. Second, at least three organizational sponsors prioritized the challenge to the extent that they provided funding and/or time, ensuring adequate resources. Finally, the timeline to implementation was 18 months to three years, avoiding pressure for immediate results while near-term enough that solutions were still relevant.

### Identifying and engaging interest holders

To identify the problem solvers to engage in solution development, we mapped interest holders impacted by the innovation challenge and any emergent solution, as well as those critical for the implementation of solutions. The group often represented a strategic microcosm of the relevant community. Engaging end users in the design process can increase the likelihood of success and accelerate the adoption of new solutions [10].

### Building a safe haven

Once interest holders were identified and invited to participate, we developed a safe haven to support collaborative productivity. A safe haven was differentiated from safe harbor in that there were no legal provisions; rather, safety was built via trust and co-creation of a shared culture [11]. Explicit and implicit steps were taken to facilitate cross-silo collaboration (see Table 1) and applied to support development of distinct environments depending upon project and interest holder needs.

### Developing a case-based learning strategy

For each innovation challenge, we developed a learning strategy anchored in case studies that provided practical, real-world considerations for solution design and implementation planning. We defined the nature and scope of cases, as well as a portfolio of cases that allowed probing of different aspects of the challenge. Cases sometimes focused on a disease, and other times on a specific product or a product class. We used historical, synthetic, or prospective cases, depending upon availability and illustrative value, as well as willingness of interest holders to share proprietary data.

Following the setup, the collaborative innovation process steps include:

1. **Elucidate challenges** – identify specific barriers to desired outcomes, including incentives, risks, and interdependencies across interest holders.
2. **Design solutions** – co-create solutions to specific identified challenges through structured dialog and interactive design.
3. **Enable implementation** – identify potential barriers and enablers to implementation of solutions created.
4. **Monitor and learn** – track uptake of solutions in real-world settings and how they evolve in practice, identifying opportunities for improvement.
5. **Disseminate and catalyze** – share learnings throughout the design process to relevant audiences to support implementation of solutions.

Table 2 summarizes application of the NEWDIGS methodology in three projects: Adaptive Licensing, Learning Ecosystems Accelerator for Patient-centered Sustainable innovation (“LEAPS”), and Financing and Reimbursement of Cures in the US (“FoCUS”). These projects were selected because they each illustrate a different type of innovation that required different strategies, indicating the diversity of application. For each project, we include an overview of the setup activities as well as steps one through three in the collaborative innovation process. Steps four and five are summarized in the Findings section.

## Findings

Three lessons relevant to the application of NEWDIGS methodology across different settings were identified. First, the methodology worked best for challenges requiring interest holders to work together to find a solution meeting everyone’s needs. It became clear that the cases most benefiting from a structured collaborative innovation process were those where diverse needs required elucidation and alignment.

Second, collaboration and development of solutions were best supported by a facilitator, an “innovation steward,” to strategically steer the setup and advance innovative collaboration. The term innovation steward has been used previously in different settings with varying definitions (e.g., [13,14]). We use it to capture a specific concept; a neutral but strategic intermediary that establishes and guides interest holder collaboration. In this context, the innovation steward enabled interactive design across interest holder silos and used the collaborative innovation process to support alignment of incentives, accelerate implementation readiness, and drive impact toward shared goals.

Third, our collective skills in collaborative health system innovation were enhanced through our work together. NEWDIGS’ experience across diverse challenges helped to advance our understanding of collaborative tools, processes, and success drivers. This capacity-building work is important as the need for collaboration to tackle complex biomedical challenges grows.

In addition to methodological lessons learned, each case study had formal and informal impacts specific to the challenge, the solution developed, and the way it was implemented. NEWDIGS’ positioning as an external innovation environment limited our insight into some of these due to proprietary constraints on shared information. Due to this only the known subsets of outcomes and impacts are discussed

### Regulatory innovation – adaptive licensing project

Adaptive Licensing, a staged approach to regulatory approval in global settings, was originally discussed as a potential solution under the name Progressive Licensing [15]. Rather than conceptualizing the model, the challenge was advancing it to readiness for evaluation in pilot activities. Implementation barriers were significant given the complex interlocking interest holder risks that would accompany change. Earlier access to medications for high-risk subpopulations of patients meant less evidence, increasing risk for regulators and payers, plus greater potential commercial risk [16]. The resulting regulatory innovation was a generalizable framework for the design and implementation of the adaptive licensing paradigm. The collaborative innovation process helped move the proposed regulatory innovation from theory into action, paving the way to a European Union pilot led by the Europeans Medicines Agency (EMA) [7].

This was the first NEWDIGS project in which collaborators recognized the value of a safe haven, multi-stakeholder, pre-competitive environment for rapid cycle learning and adaptation of innovative solutions. It inspired the launch of a new consortium (ADAPT-SMART) within the Innovative Medicines Initiative (IMI) to accelerate generalizable learnings from the EMA’s pilot project on Adaptive Licensing (rebranded at Adaptive Pathways) [17,18]. In the USA, NEWDIGS leadership also tracked related policy innovation in the 21^st^ Century Cures Act [19] and were Expert Advisory Committee participants on special report by the President’s Council of Advisors on Science and Technology [20].

### Clinical innovation – Adaptive Point-of-Care platform

NEWDIGS built on the historic precedent for clinical point-of-care data collection, analysis, and integration into decision-making [21] by facilitating the engagement in the Adaptive Point-of-Care (APoC) design process. Based on this work, a pilot of APoC use in Rheumatoid Arthritis was proposed to enable scalable evidence generation for regime optimization. Results were disseminated via peer-reviewed publication [9] and the clinical innovation functioned as a learning health system strategy influencing subsequent research design efforts led by our collaborators.

### Business innovation – orphan reinsurance and benefit manager model

Cell and gene therapies face reimbursement challenges due to high costs and clinical uncertainty. One of several new payment models developed by interest holders at NEWDIGS included Orphan Reinsurer Benefit Managers (ORBM), designed to address the financial risks of high cost, potentially curative cell and gene therapies for small insurance companies and self-insured employers. Careful innovation stewardship was required in guiding interest holders to determine the scope of implementation planning work. While some interest holders wanted to co-develop a business plan within NEWDIGS, others felt that this might constrain adoption to a single interest holder group. Ultimately, the decision was to stop short of a business plan and instead to allow the marketplace to adopt and adapt the ORBM model.

## Discussion

Biomedical science is advancing rapidly, and collaborative innovation is essential to enhancing our ability to translate these advancements into health impact. NEWDIGS’ methodology provides a structure and process that could be adapted for use across a range of innovations. NEWDIGS is known for blending key elements to enhance capacity for innovations that require system change for success:

- external innovation environment for collaboration across organizations and interest holders,
- safe haven for fostering pre-competitive collaboration,
- neutral third party serving as innovation steward, and
- proven structured methodology to enable interactive design.

For those interested in leading or stewarding cross-functional innovation environments, it is important to know that the setup and the collaborative innovation process are critically important to successful solution development. Together they push the boundaries of multi-interest holder collaboration to drive meaningful collective impact.

Organizational leaders may consider whether particular innovation efforts are more likely to succeed within an internal or external environment. By sharing our experience, we hope to enhance understanding of types of innovation challenges that might benefit from a multi-interest holder approach within an external safe haven. The details provided may also help to inform assessment of conditions under which collaboration value may outweigh proprietary risk.

The biggest limitation of this work is that to date our application of the methodology has been narrowly focused on addressing system barriers to the appropriate and timely real-world use drug therapies. In the future, application could be expanded to include life science products (e.g., diagnostics and medical devices), integration of new technical tools and capabilities into healthcare (e.g., digital health), and future state systems (e.g., clinical care and public health). It could also be expanded to other fields that would benefit from a structured and stewarded pathway to collaborative innovation.

Another limitation is the absence of a framework for measuring collective impact. We have recently begun to develop a metrics framework, involving interest holders to ensure metrics are meaningful to them and capture relevant information, including costs as well as health outcomes [22]. The framework will provide generalized principles that will be tailored to project context and goals.

Our capacity for scientific innovation far outpaces that of healthcare system innovation, and there are a growing number of transformational biomedical products that are entering a market that is unprepared for them. We believe that our collaboration methodology is a dynamic way to address complex challenges in healthcare at a time of tremendous opportunity. While its use has been focused on biomedical products, its potential to provide a platform for innovative solution design could have a much broader reach.
